# Two-carbon metabolites, polyphenols and vitamins influence yeast chronological life span in winemaking conditions

**DOI:** 10.1186/1475-2859-11-104

**Published:** 2012-08-08

**Authors:** Helena Orozco, Emilia Matallana, Agustín Aranda

**Affiliations:** 1 Departamento de Biotecnología, Instituto de Agroquímica y Tecnología de Alimentos-CSIC, Av. Agustín Escardino, 7, Paterna, 46980, Spain; 2 Departament de Bioquímica i Biologia Molecular, Universitat de València, c/Dr Moliner 50, Burjassot, 46100, Spain

**Keywords:** Wine, Yeast, Aging, Ethanol, Acetaldehyde, Resveratrol, Nicotinamide

## Abstract

**Background:**

Viability in a non dividing state is referred to as chronological life span (CLS). Most grape juice fermentation happens when *Saccharomyces cerevisiae* yeast cells have stopped dividing; therefore, CLS is an important factor toward winemaking success.

**Results:**

We have studied both the physical and chemical determinants influencing yeast CLS. Low pH and heat shorten the maximum wine yeast life span, while hyperosmotic shock extends it. Ethanol plays an important negative role in aging under winemaking conditions, but additional metabolites produced by fermentative metabolism, such as acetaldehyde and acetate, have also a strong impact on longevity. Grape polyphenols quercetin and resveratrol have negative impacts on CLS under winemaking conditions, an unexpected behavior for these potential anti-oxidants. We observed that quercetin inhibits alcohol and aldehyde dehydrogenase activities, and that resveratrol performs a pro-oxidant role during grape juice fermentation. Vitamins nicotinic acid and nicotinamide are precursors of NAD^+^, and their addition reduces mean longevity during fermentation, suggesting a metabolic unbalance negative for CLS. Moreover, vitamin mix supplementation at the end of fermentation shortens CLS and enhances cell lysis, while amino acids increase life span.

**Conclusions:**

Wine *S. cerevisiae* strains are able to sense changes in the environmental conditions and adapt their longevity to them. Yeast death is influenced by the conditions present at the end of wine fermentation, particularly by the concentration of two-carbon metabolites produced by the fermentative metabolism, such as ethanol, acetic acid and acetaldehyde, and also by the grape juice composition, particularly its vitamin content.

## Background

The *Saccharomyces cerevisiae* growth cycle during grape juice fermentation involves a growth phase, a stationary phase and a death phase [1,2]. Most sugars are consumed when cells have stopped dividing, and the death phase is usually three or four times longer than the growth phase. Therefore, yeast viability and vitality in final fermentation stages are key factors for successful winemaking. Cell death leads not only to loss of cell integrity, but also to the release of cell contents which could influence the growth of other microorganisms, such as lactic acid bacteria and spoilage yeasts [3]. Aging on lees is an enological practice involving aging in the presence of death yeasts which confers wine chemical and color stability [4]. Cell lysis has been studied in detail for sparkling wines [5], but it has been largely overlooked in primary fermentations. The yeast death phase during winemaking is still a poorly understood process, which has been exclusively linked to toxicity caused by the high ethanol concentration reached during fermentation [6].

Molecular causes of aging have been thoroughly studied in laboratory yeast strains [7]. Chronological life span (CLS) is measured as the survival of yeast cells in the stationary phase [7,8], and it is highly variable in natural isolates, including commercial wine yeast strains [9], and tends to be shorter than in laboratory strains [10]. Stress tolerance, particularly oxidative stress tolerance, is a key factor for CLS [7]. According to the traditional “free radical theory of aging”, a decline in cell functions with aging is the result of an accumulation of altered molecules generated by the effect of free radicals [11]. Reactive oxygen species (ROS), such as superoxide, have a negative impact on CLS, and antioxidant enzymes, like superoxide dismutases, are essential for extending life span [12]. Better tolerance to oxidative stress is another important factor for yeast longevity under winemaking conditions [9]. Therefore, the antioxidants that scavenge free radicals can offer benefits for life span, which was the case of the grape polyphenol quercetin under laboratory growth conditions [13].

Recently, acidification of the growth medium by acetic acid produced by the yeast metabolism has been identified as a pro-aging agent under laboratory conditions [14]. However under winemaking conditions, the impact of acetic acid on aging is prevented by grape juice’s highly buffered nature [15]. Another metabolite with an important role in aging is ethanol, which has a negative impact on the CLS of laboratory strains [16]. In such conditions, ethanol consumption by alcohol dehydrogenase 2 is controlled by the deacetylase Sir2. Sir2 is a protein whose role in aging is relevant, which has also been related for its ability to promote genome stability [7]. Sir2 uses NAD^+^ as a substrate, so it works as a cell metabolic status sensor. During the reaction, it produces nicotinamide, which acts as an inhibitor of its deacetylase activity [17]. During aerobic growth, *S. cerevisiae* can synthesize NAD^+^ from tryptophan, but it has to use the vitamins nicotinamide or nicotinic acid as precursors in the absence of oxygen [18]. Sir2 gives its name to a family of enzymes called sirtuins which, in yeast, includes four other members, Hst1 to Hst4. We have seen that these enzymes play positive and negative roles in CLS in wine yeast, and that these roles depend on the nature of the growth media [15]. Grape polyphenol resveratrol has been linked to sirtuin activation and life span extension, although this topic is the center of bitter controversy [19].

In this work, we aim to study the effect of different physical and chemical environmental factors on wine yeast CLS. We wish to know if the commercially used strains of *S. cerevisiae* react in the same way to the external factors already described for laboratory strains, and to also identify new relevant aspects for the industrial use of yeast. We have identified acetaldehyde as a potent pro-aging agent, and we have determined that polyphenols quercetin and resveratrol have an unexpected negative effect on yeast longevity, and that vitamins induce death and lysis at the end of grape juice fermentation.

## Results

### Physical factors affecting wine yeast life span

To gain a better understanding of the effect of the changes occurring in the physicochemical environment in wine yeast CLS, we tested the effect of variations in temperature, osmolarity and pH on chronological aging. To go about this, we performed standard CLS assays under laboratory conditions in synthetic complete medium SC [8]. Cells grown in SC were diluted and plated, taking day 3 of growth as time 0. In such conditions, cells reached saturation, consumed all glucose present in the medium (data not shown) and entered the stationary phase. Therefore, the death profile is represented by taking this time point as 100% viability (see Additional file 1: Table S1 for the initial cell viabilities for this and other experiments). We tested a widely used commercial strain, EC1118, whose CLS is short [9].

**Figure 1:**
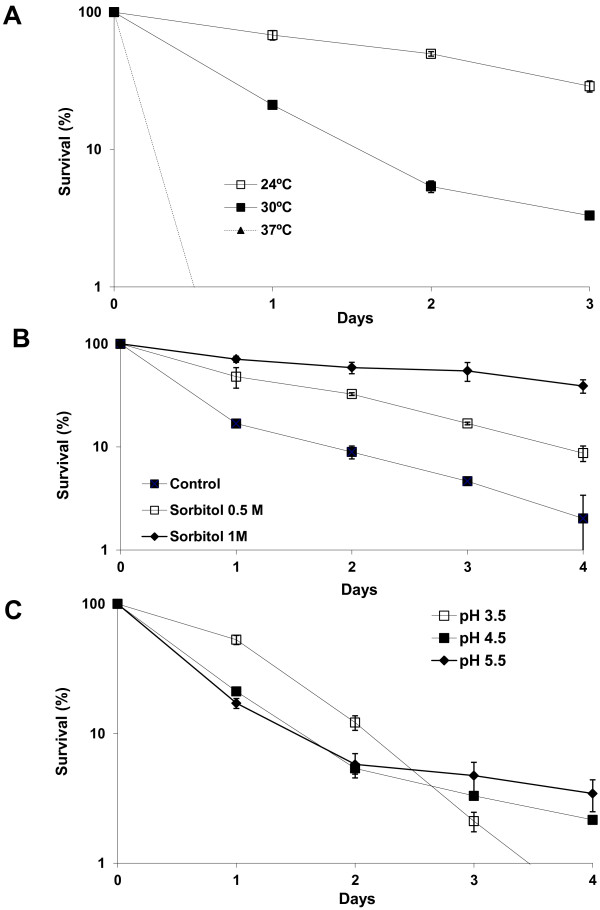
**Physical determinants of the wine yeast life span.** The CLS analyses of strain EC1118 in SC medium were done. The assays were performed by making serial dilutions, plating an aliquot and counting colonies. CFU (colony-forming units)/mL were expressed in relation to the values obtained from the day-3 post-inoculation, thatwere considered to be 100% survival. **A**) Effect of temperature on CLS. **B**) Effect of hyperosmorality on CLS. **C**) Effect of pH on CLS. Experiments were done in triplicate, and the mean and standard deviation are provided.

First we analysed the effect of temperature on life span (Figure 1A). A culture was split at time 0 and the aliquots were incubated at three different temperatures within the range of temperatures that yeast may encounter during their industrial use. We included 30°C as a standard laboratory condition, and also a lower (24°C) and a higher (37°C) temperature. We found that both the mean (50% viability) and maximum CLS shortened as temperature increased, and that raising temperatures to 37°C had a dramatic effect on longevity, which dropped to values of below 1% viability in just one day (Figure 1A). Lowering the temperature to 24°C greatly increased viability, therefore lower fermentation temperatures help to maintain cell viability.

Next we tested the effect of osmotic pressure on CLS by adding increasing amounts of osmolyte sorbitol to the growth medium (Figure 1B). Compared to the control experiments without sorbitol, increasing medium osmolarity by adding 0.5 M sorbitol extended the mean and maximum life spans, while the addition of 1 M sorbitol further prolonged it. Thus, increasing osmolarity extends the wine yeast life span. Moreover, 1 M sorbitol mimics a 20% glucose concentration [20], an environment in which wine strains have evolved. An experiment with a SC medium with a 20% glucose concentration led to a dramatic loss of viability (data not shown), probably due to the high ethanol concentration produced. Therefore hyperosmotic stress extends longevity, but thermal stress shortens it, suggesting that these environmental signals trigger different pathways that affect survival.

Finally, we tested survival in media with different pHs (Figure 1C). The pH of our SC medium was 4.5. We tested higher (5.5) and lower (3.5) pH values. The higher pH 5.5 gave a fairly similar CLS profile to pH 4.5, although it slightly extended maximum survival. The lower pH 3.5 was in the range found in natural grape juices, and gave a different death profile. Mean life span survival increased, but viability dropped faster leading to a shortened maximum life span. Therefore, distinct physical stimuli affect the death profile differently.

### Two-carbon metabolites reduce wine yeast CLS

**Figure 2:**
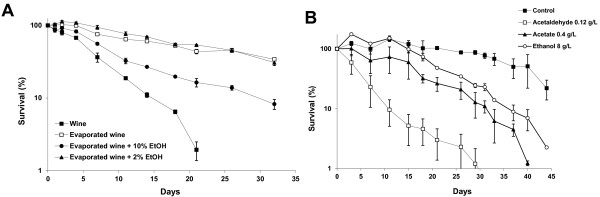
**Role of two-carbon metabolites in the wine yeast life span.A**) The CLS analyses were performed with strain EC1118 from a completed fermentation in synthetic grape juice placed in another completed fermentation (wine), from which ethanol was removed by evaporation (evaporated wine), and with evaporated wine containing 2% and 10% ethanol. The time when cells were placed in the new medium is considered 0. **B**) CLS analysis of the T73 strain in water containing ethanol (8 g/L), acetate (0.4 g/L) and acetaldehyde (0.12 g/L). The assays were performed as described in Figure 1. Experiments were done in triplicate, and the mean and standard deviation are provided.

As ethanol is the main stress condition at the end of fermentation, and since it has been considered to be the main one and sometimes the only cause of death during grape juice fermentation [6], we studied the impact of ethanol on life span once fermentation had been completed in more detail. To this end, fermentation on synthetic grape juice MS300 [21] was performed. Once the sugars had been consumed, cells were spun and the supernatant was taken as a control (“wine”). Ethanol was evaporated from part of this medium, dropping from 10.7% to 0.6% (wt/vol) to obtain an “evaporated wine”. Ethanol was added at concentrations of 2% and 10% (the latter restored the initial concentration) to the “evaporated wine”. An independent fermentation on the same synthetic grape juice was carried out with strain EC1118. Once the sugars had been completely consumed after 9 days of growth, 30-mL aliquots were centrifuged and cells resuspended in the same volume of the various already prepared media, and viability was measured over time by diluting, planting and counting the colonies grown in the rich medium (Figure 2A). Firstly, it is worth noting that evaporation indeed extended CLS greatly, thus confirming that ethanol is a key player in longevity at the end of grape juice fermentation. However, it is not the only one as yeast cells in evaporated wine + 10% ethanol showed an extended life span despite having the same amount of ethanol. Hence, other volatile compounds produced during fermentation were also important for longevity (see below). The addition of a small amount of ethanol (evaporated wine + 2% ethanol) did not affect CLS, indicating that only a large amount of ethanol affects wine yeast longevity under winemaking conditions (0.8% ethanol shortened CLS under laboratory conditions [16]). In fact at the beginning of the aging experiments, the culture grown in evaporated wine + 2% ethanol grew slightly; i.e., it reached 113% survival after day 2 (note the logarithmic scale of Figure 2) and, before day 7, cell viability was over 100%. In general, these findings indicate that once fermentation has been completed, cells can adapt to a new environment, indicating that cells are not programmed to necessarily die at the end of fermentation when all the sugars have been consumed.

The previous experiments suggest that volatile compounds, in addition to ethanol, are relevant for yeast aging during winemaking. For laboratory strains, it has been described that acetic acid [14] shortens CLS. We aimed to study the effect on yeast longevity of acetate and acetaldehyde (the metabolic intermediary between ethanol and acetate) at the concentrations found after grape juice fermentation in wine. To go about this, yeast was grown for three days in SC as previously described. Then cells were washed and resuspended in water to eliminate the presence of any metabolite in the exhausted medium. Aliquots were split, the two-carbon metabolites were added and viability was followed over time (Figure 2B). Our laboratory reference wine strain T73, which has an intermediate CLS [9], was used. Incubation in water mimics extreme starvation that extends CLS ( [8]; Figure 2). 8 g/L ethanol, which shortens CLS in laboratory strains, also shortened longevity in wine yeast. Potassium acetate was employed to avoid the effect of changes in extracellular pH. Moreover, 0.4 g/L of acetate greatly affected CLS, even to a greater extent than an amount of ethanol that is 20 times *larger.* This confirms that acetate is a strong pro-aging factor which also affects wine yeast. Nevertheless, a stronger effect on longevity was obtained with acetaldehyde, which was achieved at an even lower concentration (0.12 g/L). The same experiment was performed using the EC1118 strain (Additional file 2: Figure S1), and again acetaldehyde had a strong pro-ageing effect, in this case similar to the one produced by ethanol. Acetate shortens CLS, although to a lower extent. This is the first time that acetaldehyde has been described as an aging inducer factor. Some of the negative effects caused on CLS by ethanol and acetate addition are probably due to their conversion into this highly reactive molecule.

### Resveratrol and quercetin have a negative impact on yeast life span during fermentation

**Figure 3:**
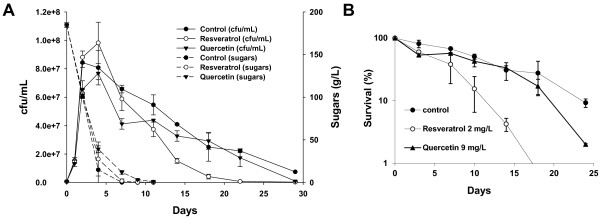
**Analysis of the effect of resveratrol and quercetin on wine fermentation.A**) Cell viability and sugar consumption of a fermentation on synthetic grape juice by strain EC1118 with the addition of resveratrol (2 mg/L) and quercetin (9 mg/L). **B**) CLS profile by taking day 4 of the fermentation described in panel A) as 100% survival. Experiments were done in triplicate, and the mean and standard deviation are provided.

To test the impact on fermentation and the yeast CLS of grape polyphenols resveratrol and quercetin, we performed fermentations with wine yeast strain EC1118 in synthetic grape juice MS300 [21], containing 2 mg/L resveratrol or 9 mg/L quercetin; both these concentrations are normally found in natural macerated red grape juice [22]. The cell viability and sugar consumption during fermentation is shown in Figure 3A. The aging profile was plotted by taking day 4 as 100% viability as this was the time point at which saturation was reached (Figure 3B). Resveratrol slightly increased the highest cell viability to induce a rapid loss of viability, which led the mean and maximum CLS to shorten quickly. Quercetin had a slight negative effect on growth, causing not only lower maximal cell density, but also a drop in CLS which was lower than the control situation during most of the fermentation, particularly at the end of it, although its effect was slighter than that observed for resveratrol (Figure 3). There was some delay in sugar consumption by the polyphenol containing fermentations, particularly in the case of quercetin, although all fermentation reached completion by day 9 (Figure 3A). Production of key metabolites such as ethanol, acetaldehyde and acetic acid was measured during fermentation (Additional file 3: Figure S2). The profile of metabolite production is similar between the fermentations, but ethanol production is reduced in the fermentation containing quercetin, while acetaldehyde production is slightly bigger at the end of the polyphenol-containing fermentations, particularly at the end. Acetic acid production is very similar. Therefore ethanol and acetic acid seem not to be the case to the shorter CLS, while acetaldehyde increase may contribute to the decrease in life span caused by these polyphenols.

**Figure 4:**
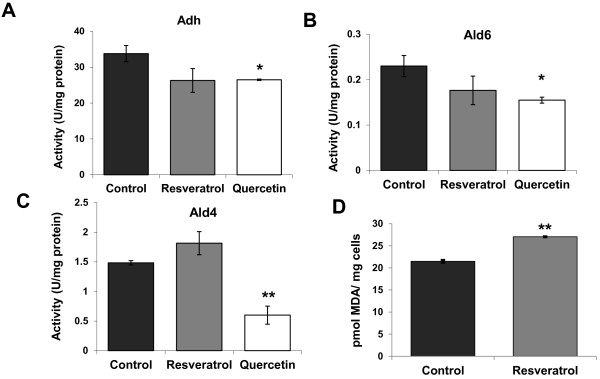
**Polyphenols influence enzymatic activity.A**) The alcohol dehydrogenase activity of wine yeast extracts in the presence of resveratrol (2 mg/L) and quercetin (9 mg/L). **B**) Cytosolyc aldehyde dehydrogenase Ald6p activity under the same conditions. **C**) Mitochondrial aldehyde dehydrogenase Ald4p activity under the same conditions. **D**) Lipid peroxidation levels on day 6 of a fermentation on synthetic grape juice with and without resveratrol. Experiments were done in triplicate, and the mean and standard deviation are provided. *p < 0.05, **p < 0.01, unpaired *t*-test, two-tailed.

Polyphenols may have an impact on the activity of several enzymes. Resveratrol and quercetin have been described to interfere with the activity of cytosolic sheep aldehyde dehydrogenase [23]. We tested the effect of these polyphenols (at the concentrations used in the previous experiment) on the activity of alcohol and aldehyde deshydrogenases, these being important enzymes in yeast metabolism and in the interplay between the two-carbon metabolites. Extracts from the yeast cells on stationary phase were assayed for different enzymatic activities (Figure 4). Alcohol dehydrogenase transformed acetaldehyde into ethanol during fermentation. Both polyphenols reduced this activity, but only quercetin did so significantly (Figure 4A). That may explain the slightly higher acetaldehyde and lower ethanol production caused by this polyphenol (see Additional file 3 Figure S2). Part of acetaldehyde can be transformed into acetic acid by aldehyde dehydrogenase. We tested the main cytosolic, K^+^ and NADP^+^-dependent activity, encoded by gene *ALD6* (Figure 4B). Once again quercetin showed significantly decreased enzymatic activity. This reduction was further evidenced in the mitochondrial, Mg + and NAD^+^-dependent enzyme *ALD4* (Figure 4C). In this case, resveratrol showed a slight, but not significant, increase in this activity. Quercetin has been proposed to occupy the NAD^+^ pocket of the ovine aldehyde dehydrogenase [23], and in yeast, it appears that this ability may cause the inhibition of those enzymes using NAD(P) ^+^ as a cofactor. The inhibitory effect of these enzymes by quercetin may lead to an increase of intracellular acetaldehyde that may be the cause of the delayed growth (see Figure 2A).

Resveratrol has been described to have both antioxidant and pro-oxidant properties depending on the concentration and the environment involved [24]. To test its impact on our working conditions, cells from day 6 of a fermentation done on synthetic grape juice (onset of the death phase; see Figure 3) with and without 2 mg/L of resveratrol were taken and its levels of lipid peroxidation, as marker of oxidative damage, were analysed (Figure 4D). Samples from the fermentation with resveratrol showed an increased oxidative damage, indicating that during fermentation, resveratrol acts as a pro-oxidant, leading probably to the decrease in CLS we observed (Figure 4B).

### Role of NAD(P)^+^ precursors on yeast life span during fermentation

**Figure 5:**
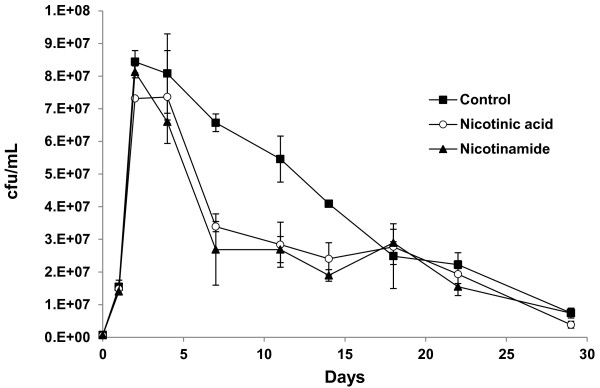
**Nicotinamide and nicotinic acid exert a negative effect during wine fermentation.** The cell viability of a fermentation on synthetic grape juice by strain EC1118 with the addition of nicotinic acid and nicotinamide at 3.75 mg/L. Experiments were done in triplicate, and the mean and standard deviation are provided.

NAD(P) is a key cofactor for metabolism and helps maintain the redox homeostasis. Besides, NAD^+^ is used as a substrate in the deacetylation reaction performed by sirtuins. Under aerobic conditions, NAD^+^ can be synthesized from tryptophan but, under anaerobic conditions, it is synthesized only from nicotinic acid or nicotinamide which, in such conditions, are considered vitamins [18]. To test the influence of these compounds on aging, we carried out fermentations in synthetic grape juice MS300, which contains only 1 mg/L of tryptophan, the minimal amount found naturally in grape juices [25], with or without 3.75 mg/L of nicotinic acid or nicotinamide, the highest concentration of nicotinic acid found in grape juice (Cantor, 1953). Nicotinamide addition had no major impact on cell growth as this fermentation reached a similar final cell density to the control one (Figure 5). The maximum cell count was slightly lower for the fermentation containing nicotinic acid (Figure 5). Viability started to fall faster during the nicotinamide-containing fermentation if compared to the control experiment, thus reducing the mean life span, and both became similar at longer times. Nicotinic acid addition produced a similar cell viability profile, but with a slightly lower maximum life span. In all cases, the metabolic rate was similar as all the strains consumed glucose at a similar rate, with fermentation finishing at around day 11 (data not shown). Therefore, cells can grow with the NAD^+^ produced from small amounts of tryptophan, and an additional source from vitamins nicotinic and nicotinamide was not required for growth, and even proved detrimental for the mean life span.

### Vitamins impact on cell lysis

**Figure 6:**
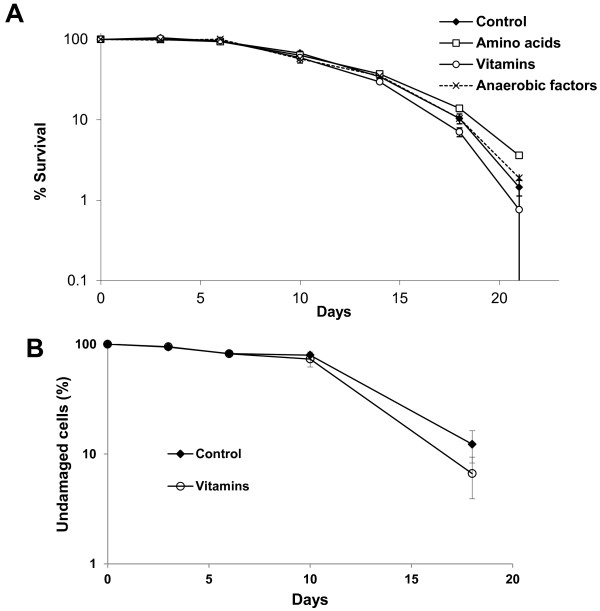
**Effect of micronutrients on life span during wine fermentation.A**) The CSM strain’s CLS profile by taking the day when sugars were consumed as 100% survival. Cell viability after adding amino acids, vitamins and anaerobic factors was followed. **B**) Cell integrity measured as propidium iodide staining was followed under the same conditions. Experiments were done in triplicate, and the mean and standard deviation are provided.

**A**fter death, cell lysis at the end of fermentation influences the final product’s properties and stability. To study the impact of nutrients from grape juice, which may have limiting amounts and affect longevity, fermentation on red grape juice was carried out with CSM, a commercial yeast strain with a long CLS [9]. When sugars had been exhausted and fermentation had ended, aliquots were split, and amino acids, vitamins and anaerobic factors were added in the amounts used in the synthetic grape juice recipe [21]. Cell viability was followed by taking aliquots, diluting, plating and counting the cfu, and the end of fermentation was taken as 100% viability. Addition of anaerobic factors (a mixture of oleic acid and ergosterol) had no impact on cell viability (Figure 6A). These compounds may help membranes to deal with ethanol stress, but it seems that they are not a limiting factor on natural grape must after fermentation has ended. Addition of a mixture of amino acids enhances maximum life span, probably because a metabolic deficiency is covered. Surprisingly, additional vitamins shortened CLS. pH was not changed by vitamin and anaerobic factors addition, and amino acid addition caused an very slight change form pH 3.20 to 3.27 that are inside the pH range of wine. Therefore, addition of amino acids may stimulate longevity and cell integrity at late stages of wine fermentation, and addition of vitamins may be a good way of stimulating cell death. To analyze the effect of vitamins on cell lysis, a similar experiment was carried out and cell integrity was analyzed with propidium iodide, which stains damaged cells [26]. Vitamin addition also stimulated cell lysis (Figure 6B) and diminished cells’ ability to divide. Therefore, vitamin supplementation is a way of stimulating cell lysis at the end of fermentation.

## Discussion

In this work, the physical and chemical environmental determinants affecting wine yeast chronological life span (CLS) were analyzed. We have determined that heat and a low pH shortened the maximum CLS, while a hyperosmotic environment extends it (see Figure 1). However, a low pH extends the mean life span. Natural grape juices have a low pH, so wine yeasts are well adapted to it, which may benefit cell survival as long as nutrients are present. Once resources have been exhausted, cells may find it harder to keep a balance with an environment that has a lower pH than the cytosol. Intracellular pH is around 5.5 in the stationary phase [27], so this may benefit long-term survival in a higher pH medium which is not so energy-demanding. Osmotic stress has been described to extend longevity in laboratory strains [28], which may be a case of stress cross protection, or hormesis, when a stress condition protects against subsequent stress conditions. Hyperosmotic stress triggers glycerol biosynthesis, a molecule that acts as an anti-aging molecule [29]. This may prove useful for winemaking conditions, although the ethanol produced by high sugar fermentation may cancel out the positive effect of glycerol because ethanol is a well-known pro-aging factor ( [16] and Figure 2).

Ethanol, along with acetic acid, another pro-aging factor identified in experiments with laboratory strains [14], are aging inducers in wine yeast strains (Figure 2B). This scenario indicates that the genetics underlying aging mechanisms is common between laboratory and industrial strains. Besides, acetaldehyde has been identified as a pro-aging factor for the first time. This molecule has a greater impact at lower doses (at the concentrations usually detected in wines undergoing regular fermentation processes), which suggests that part of the ethanol and acetic acid impact on longevity may be due to rapid conversions between the two-carbon metabolites during fermentative growth. We previously found that acetaldehyde represses the genes involved in cell cycle progression [30]. Under enological conditions, ethanol was traditionally thought to be the main cause of aging; indeed, its removal greatly extends CLS after fermentation completion (Figure 2A). However, there are other compounds, acetic acid and acetaldehyde, which contribute to longevity as added ethanol has only a partial negative effect on CLS. Cells can adapt to a change in the environment once sugars have been exhausted (Fig 2A). This circumstance suggests that the ability to adapt a metabolism is a key factor in determining lifespan, and that yeasts are not strictly bound to dye after sugar exhaustion. At the end of fermentation, we saw damaged cells stained by propidium iodide (Figure 6B), a marker of necrosis, suggesting that cells may die and lyse due to the accumulation of these toxic two-carbon compounds generated during fermentation.

We tested the effect of two polyphenols commonly found in grapes, resveratrol and quercetin, on CLS during fermentation. Both molecules exert a negative effect on cell viability. Resveratrol is a phytoalexin produced by the grape to prevent fungal infections, and it does act as an antifungal compound against *S. cerevisiae*[31], what may explain its negative impact on life span. Resveratrol has been shown to have both antioxidant and pro-oxidant effects [24]. For instance, in the presence of copper ions, resveratrol acts as a pro-oxidant agent by affecting DNA [32]. With our experimental conditions, resveratrol causes increased lipid peroxidation damage (Figure 4D), a well-known oxidative stress marker, indicating that resveratrol causes internal oxidative stress which may compromise cell viability. Quercetin is regarded as an antioxidant with a positive effect on CLS under laboratory conditions [13]. However, we noted the opposite effect for winemaking conditions. We have shown that quercetin has a negative effect on a variety of wine yeast enzymatic activities, aldehyde and alcohol dehydrogenases (Figure 5), probably due to its ability to compete with NAD(P)^+^ in some enzymes, such as aldehyde dehydrogenase [23]. This inhibition may prove more relevant during grape juice fermentation than during growth on laboratory media, leading to a high intracellular acetaldehyde concentration that may be deleterious for the cell (note the higher final acetaldehyde concentration in Figure 2B). Resveratrol may also affect other enzymes in a similar manner. It has been described to stimulate sirtuins [33], key enzymes in life span control, by extending the replicative life span. If this were the case, then our results would indicate that sirtuin activity is negative for CLS under winemaking conditions. We have previously shown that some sirtuins, like Sir2, play a positive role during grape juice fermentation, while others, like Hst2 and Hst3, perform a negative one [15]; therefore, the latter may be more relevant for CLS in fermentation than the former. Indeed, this would fit in with the fact that the addition of vitamins nicotinic acid and nicotinamide, which produce more NAD^+^ and more sirtuin activity, also had a negative effect on CLS. Alternatively, excess NADH to be re-oxidized during fermentation could produce a metabolic unbalance, which may affect cell performance and viability.

Modulating cell death and subsequent lysis are important features that have been studied in the secondary fermentation of sparkling wines, but not in detail during primary fermentation. We identify herein some environmental factors that can be modulated to stimulate or prevent death and cell breakage. The most promising intervention is vitamin supplementation. We observed how the addition of vitamins of group B2 (nicotinic acid and nicotinamide) at the beginning of fermentation shortens the mean cell life span (Figure 5), and how the addition of a mixture of vitamins at the end of fermentation stimulates cell lysis (Figure 6). Currently, we do not understand the molecular reason for these effects and further research is required to see the full picture of cell death and lysis after grape juice fermentation.

## Conclusions

Wine *S. cerevisiae* strains are able to sense changes in the environmental conditions and alter their chronological life span, both extending or shortenning it. When sugars are completely consumed and therefore wine fermentation has been completed, yeasts are not bound to die and they are able to adapt to changes in the media composition. For instance, lowering the ethanol concentration or adding amino acids at this stage extend yeast life span, while vitamin addition shortens it. Yeast death is influenced by the growth medium composition, but also by the conditions present at the end of wine fermentation, particularly by the concentration of two-carbon metabolites produced by the fermentative metabolism, such as ethanol, acetic acid and acetaldehyde (a chemical that has been identified as a pro-aging factor in this work). Surprisingly the presence of polyphenols generally regarded as antioxidants, such as resveratrol and quercetin, has a negative effect on yeast viability during grape juice fermentation.

## Methods

### Yeast strains and growth media

Commercial wine yeast strains EC1118, CSM and T73 were a gift from Lallemand Inc. (Toronto, Canada). For yeast growth under laboratory conditions, YPD medium (1% yeast extract, 2% bactopeptone, 2% glucose) and SC medium (0.17% yeast nitrogen base, 0.5% ammonium sulfate, 2% glucose and 0.2% drop-out mix with all the amino acids) were used [34]. SC pH was adjusted with HCl or NaOH to obtain different pH values, and sorbitol was added at 0.5 or 1 M to increase osmolarity. Synthetic grape juice MS300 was prepared as described by Riou et al. [21], but with an equimolar amount of glucose and fructose at 10%. In addition to the carbon source it contains malic acid 6 g/L, citric acid 6 g/L, assimilable nitrogen source 300 mg N/L (120 mg as (NH_4_)Cl and 180 mg as amino acids), mineral salts (KH_2_PO_4_ 750 mg/L, K_2_SO_4_ 500 mg/L, MgSO_4_ 250 mg/L, CaCl_2_ 155 mg/L, NaCl 200 mg/L), oligoelements, vitamins (myo-inositol 20 mg/L, calcium panthothenate 1.5 mg/L, nicotinic acid 2 mg/L, chlorohydrate thiamine 0.25 mg/L, chlorohydrate pyridoxine 0.25 mg/L, biotine 0.003 mg/L), and anaerobic factors (ergosterol 15 mg/L and oleic acid 5 mg/L, Tween 80 0.5 ml/L) at pH 3.3. Red grape juice (Bobal variety) was a gift from Bodegas Murviedro and was sterilized overnight with 500 μg/L of dimethyldicarbonate.

### Yeast growth conditions and chronological life span measurements

Under laboratory conditions, the CLS experiments were adapted from Fabrizio and Longo [8], and were performed as follows: the precultures of selected strains were grown overnight on YPD and inoculated in SC media (and derivatives) at an OD_600_ of 0.1. From day 3 of growth at 30°C, aliquots were taken, diluted and plated. Colonies were counted and the percentage of survival was calculated by taking day 3 of growth as 100% survival. To test the effect of two-carbon metabolites, cells at day 3 were spun, washed and resuspended in water containing the desired amount of each metabolite, and aging was performed as previously described.

For the microvinification experiments, cells from 2-day cultures in YPD were inoculated at a final concentration of 10^6^ cells/mL in filled-in loosely closed conical centrifuge tubes with 50 mL of grape juice. Incubation was performed with very low shaking at 22°C for natural juice and at 24°C for synthetic grape juice. Evolution of vinifications was followed by determining cell viability and sugar consumption, as previously described [35]. Consumption of sugars during fermentation was measured by their reaction to DNS (dinitro-3.5-salycilic acid) following a modified version of Miller’s method [36].

### Enzymatic activities

To measure enzymatic activities, cells extracts were prepared under the previously described conditions [37], but by breaking cells with one volume of glass beads in a Fast-Prep 24 (MP Biomedicals). Enzymatic activity from alcohol dehydrogenase and aldehyde dehydrogenase activities were performed according to Postma (1989) and to a previous work [38], respectively. Protein concentration was measured by the Bio-Rad Protein Assay following the manufacturer’s instructions.

### Lipid peroxidation determinations

Quantification of lipid peroxidation was carried out by the reaction of thiobarbituric acid with the malondialdehyde (MDA) product of oxidized fatty acid breakage [39]. Cells were collected and then extracted by vortexing with one volume of glass beads in 0.5 ml of 50 mM sodium phosphate buffer, pH 6.0, 10% trichloroacetic acid (TCA) with FastPrep 24. After centrifugation, 300 μL of supernatants were mixed to 100 μL of 0.1 M EDTA and 600 μL 1% thiobarbituric acid in 0.05 M NaOH to be then incubated at 100°C for 15 min. Malondialdehyde was measured by absorbance at 535 nm.

### Microscopy methods

For propidium iodide staining, 500 μl of cells were washed in PBS buffer and 5 μl of a 1 mg/mL stock solution of the dye were added to be then incubated in darkness for 30 min. Cells were washed in PBS and visualized. Cells were viewed after a 2-hour incubation at room temperature in the darkness. Cells were visualized with a rhodamine filter under a Nikon eclipse 90i fluorescence microscope.

## Competing interests

The authors declare that they have no competing interests.

## Author’s contributions

HO carried out the experimental methods. EM contributed to the experimental design. AA designed the experiments and wrote the manuscript. All authors discussed the data, and read, reviewed and approved the final manuscript.

## Supplementary Material

Additional file 1 **Table S1.** Initial viability of the chronological life span experiments (in cfu/mL x10^6^) Click here for file

Additional file 2 **Figure S1.** CLS analysis of the EC1118 strain in water containing ethanol (8 g/L), acetate (0.4 g/L) and acetaldehyde (0.12 g/L). The assays were performed as described in Figure 1. Experiments were done in triplicate, and the mean and standard deviation are provided. Click here for file

Additional file 3 **Figure S2.** Production of ethanol (A), acetaldehyde (B) and acetic acid (C) during the grape juice fermentation in synthetic grape juice containing 2 mg/L resveratrol or 9 mg/L quercetin described in Figure 2A. Experiments were done in triplicate, and the mean and standard deviation are provided. Click here for file
