# Supplementary material for: Two-carbon metabolites, polyphenols and vitamins influence yeast chronological life span in winemaking conditions
Source: Microb Cell Fact. 2012 Aug 8;11:104. doi: 10.1186/1475-2859-11-104 (PMC3503821; doi:10.1186/1475-2859-11-104)
Supplement: Additional file 1 — Table S1. Initial viability of the chronological life span experiments (in cfu/mL x106) [file 1475-2859-11-104-S1.pdf]

Table S1. Initial viability of the chronological life span experiments (in cfu/mL x10^6)

Figure	Treatment	cfu/mL x10^6
**Figure 1A**	All temperatures	90.77
**Figure 1B**	Control	140.37
	Sorbitol 0.5 M	108.43
	Sorbitol 1 M	64.63
**Figure 1C**	pH 3.5	73.33
	pH 4.5	66.77
	pH 5.5	86.33
**Figure 2A**	Wine	24.75
	Evaporated wine	25.55
	Evaporated wine + 10% EtOH	23.85
	Evaporated wine + 2% EtOH	23.80
**Figure 2C**	All conditions	50.33
**Figure 6A**	All conditions	66.60
