# Supplementary figures and images for: Two-carbon metabolites, polyphenols and vitamins influence yeast chronological life span in winemaking conditions
Source: Microb Cell Fact. 2012 Aug 8;11:104. doi: 10.1186/1475-2859-11-104 (PMC3503821; doi:10.1186/1475-2859-11-104)

Figure S1

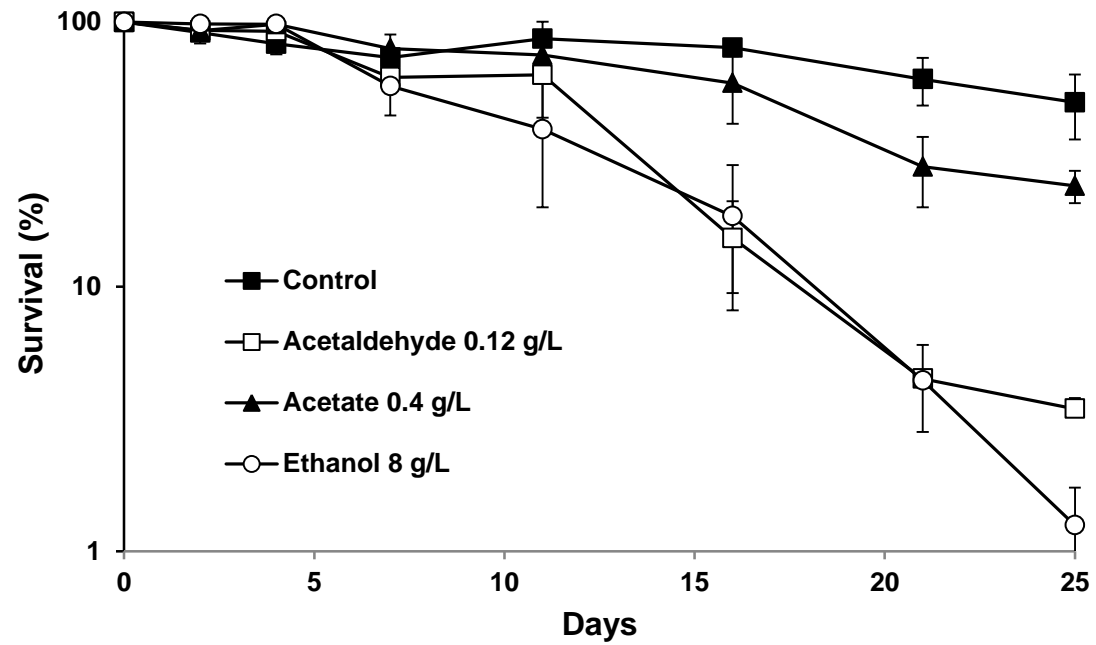

Supplement: Additional file 2 — Figure S1. CLS analysis of the EC1118 strain in water containing ethanol (8 g/L), acetate (0.4 g/L) and acetaldehyde (0.12 g/L). The assays were performed as described in Figure 1. Experiments were done in triplicate, and the mean and standard deviation are provided. [file 1475-2859-11-104-S2.pdf]

**A**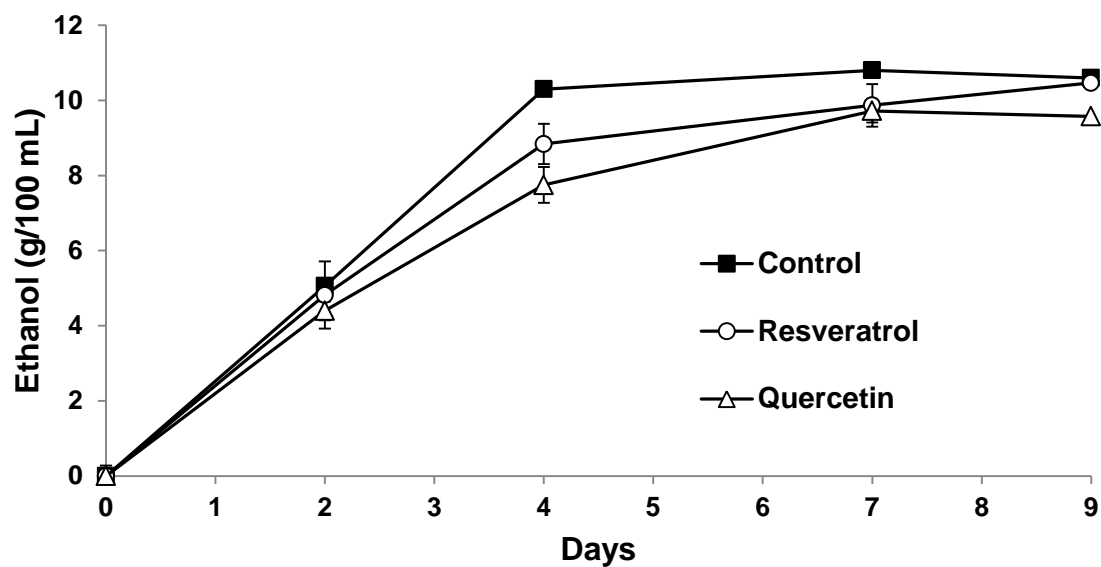**B**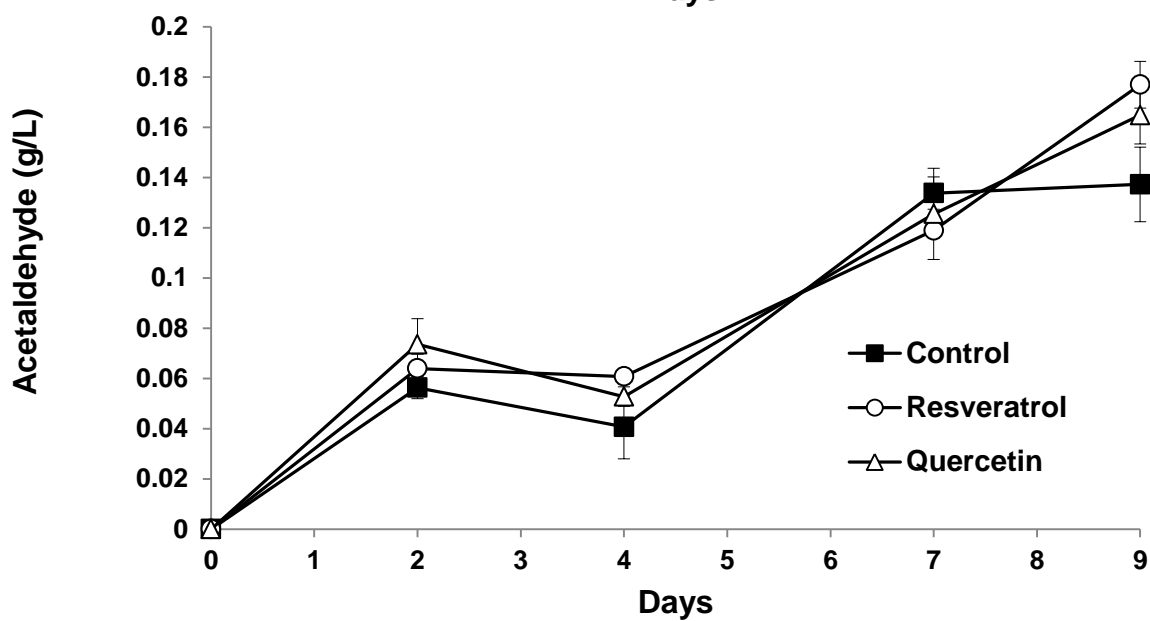**C**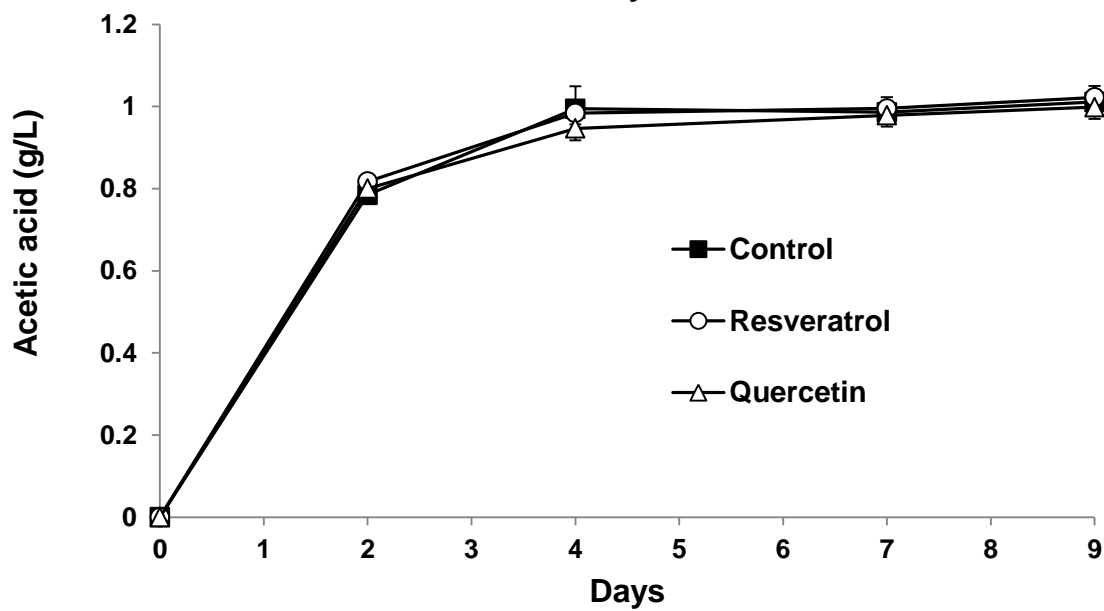

Supplement: Additional file 3 — Figure S2. Production of ethanol (A), acetaldehyde (B) and acetic acid (C) during the grape juice fermentation in synthetic grape juice containing 2 mg/L resveratrol or 9 mg/L quercetin described in Figure 2A. Experiments were done in triplicate, and the mean and standard deviation are provided. [file 1475-2859-11-104-S3.pdf]
